# Oxaliplatin induces different cellular and molecular chemoresistance patterns in colorectal cancer cell lines of identical origins

**DOI:** 10.1186/1471-2164-14-480

**Published:** 2013-07-16

**Authors:** Piroska Virag, Eva Fischer-Fodor, Maria Perde-Schrepler, Ioana Brie, Corina Tatomir, Loredana Balacescu, Ioana Berindan-Neagoe, Bogdan Victor, Ovidiu Balacescu

**Affiliations:** 1 The Oncology Institute “Prof.Dr.I. Chiricuta”, 400015 Republicii Str., nr. 34-36, Cluj-Napoca, Romania; 2 University of Medicine and Pharmacy “Iuliu Hatieganu”, 400012 Victor Babes Str., nr. 8, Cluj-Napoca, Romania

**Keywords:** Oxaliplatin, Colorectal cancer, Chemoresistance, DNA cross-links, Gene expression profile

## Abstract

**Background:**

Cancer cells frequently adopt cellular and molecular alterations and acquire resistance to cytostatic drugs. Chemotherapy with oxaliplatin is among the leading treatments for colorectal cancer with a response rate of 50%, inducing intrastrand cross-links on the DNA. Despite of this drug’s efficiency, resistance develops in nearly all metastatic patients. Chemoresistance being of crucial importance for the drug’s clinical efficiency this study aimed to contribute to the identification and description of some cellular and molecular alterations induced by prolonged oxaliplatin therapy. Resistance to oxaliplatin was induced in Colo320 (Colo320R) and HT-29 (HT-29R) colorectal adenocarcinoma cell lines by exposing the cells to increasing concentrations of the drug. Alterations in morphology, cytotoxicity, DNA cross-links formation and gene expression profiles were assessed in the parental and resistant variants with microscopy, MTT, alkaline comet and pangenomic microarray assays, respectively.

**Results:**

Morphology analysis revealed epithelial-to-mesenchymal transition in the resistant vs parental cells suggesting alterations of the cells’ adhesion complexes, through which they acquire increased invasiveness and adherence. Cytotoxicity measurements demonstrated resistance to oxaliplatin in both cell lines; Colo320 being more sensitive than HT-29 to this drug (*P* < 0.001). The treatment with oxaliplatin caused major DNA cross-links in both parental cell lines; in Colo320R small amounts of DNA cross-links were still detectable, while in HT-29R not. We identified 441 differentially expressed genes in Colo320R and 613 in HT-29R as compared to their parental counterparts (at least 1.5 -fold up- or down- regulation, p < 0.05). More disrupted functions and pathways were detected in HT-29R cell line than in Colo320R, involving genes responsible for apoptosis inhibition, cellular proliferation and epithelial-to-mesenchymal transition. Several upstream regulators were detected as activated in HT-29R cell line, but not in Colo320R.

**Conclusions:**

Our findings revealed a more resistant phenotype in HT-29R as compared to Colo320R and different cellular and molecular chemoresistance patterns induced by prolonged treatment with oxaliplatin in cell lines with identical origins (colorectal adenocarcinomas).

## Background

The last decade has brought major improvements in the treatment of cancers, but in spite of the efficacy of the cytostatic drugs, in time, cells adopt several cellular and molecular alterations, acquiring resistance. In colorectal cancer (CC) the 5 years survival rate remains lower than 10% in patients with metastasis, mainly due to resistance to the cytostatic drugs [1], regardless of the use of targeted molecular therapies in addition to standard chemotherapeutic regimens. The major treatment for metastatic CC is represented by 5-fluorouracil (5FU) and oxaliplatin (L-OHP). While 5FU inhibits thymidylate synthase during DNA replication [2], L-OHP acts as a bifunctional alkylating agent, covalently binding DNA and forming platinum-DNA adducts [3]. The intrastrand cross-links formed by L-OHP being the most abundant lesions capable of blocking both replication and transcription of DNA, they are considered to cause the major cytotoxic lesions and being directly involved in the cancer cells death [4,5].

L-OHP [(1R, 2R)-cyclohexane-1, 2-diamine] (ethanedioato-O, O’) platinum (II), a third generation platinum analogue, is the first compound that have proved to be efficient in the treatment of CC in patients displaying resistance to cisplatin (CDDP) and carboplatin (CBCDA) [6]. Although the response rate to current systemic therapies is 50% resistance develops in almost all patients [7], limiting the drug’s therapeutic potential. Cells become resistant to platinum-based drugs through reduced cellular uptake, impaired DNA adducts formation, alterations in DNA repair genes such as ERCC1 and XRCC1 and modifications in the levels of copper transporters (ATP7A and ATP7B) [8-10].

Although in the last decade the gene expression profiling of human cancer cells provided valuable insight into the molecular targets of chemoresistance, the mechanisms involved in L-OHP resistance of CC are still poorly understood and the cellular and molecular alterations are not completely recognized.

Our study proposed to identify and describe some of the cellular and molecular alterations that occurred in CC cell lines with induced chemoresistance to L-OHP. In an earlier study, aiming to evaluate the differences in the behavior of the cells selected for L-OHP resistance compared to the sensitive ones, we assessed the cytotoxicity, apoptosis and induction of DNA damages by L-OHP in Colo320 CC cell line. We found lower toxicity, cellular death and fewer DNA damages, in the cells treated previously with L-OHP as compared to the parental ones [11]. In the present study we performed a comparative study on two CC cell lines (Colo320 and HT-29) with identical origins (adenocarcinomas) and their L-OHP resistant counterparts (Colo320R and HT-29R) obtained by prolonged exposure to L-OHP. In addition, we analyzed the cells’ morphological features, DNA cross-links formation and the gene expression profiles. The DNA cross-links induction by L-OHP was determined indirectly with alkaline comet assay (CA), by introducing single strand breaks via ionizing radiation. The reduction of the single strand-breaks produced by the ionizing radiation in the cells treated with L-OHP quantitatively reflected the cross-links induced by the platinum compound. Gene expression profiling and subsequent validations with quantitative real-time PCR (qRT-PCR) were conducted in order to identify the molecular targets and pathways altered during chemoresistance acquirement. Finally, a comparative study was performed between the functions and pathways modulated by L-OHP treatment in the tested cell lines, in order to identify some common patterns of chemoresistance in CCs.

This study demonstrated that prolonged treatment with L-OHP induces several cellular and molecular targets and pathways, which lead to chemoresistance, but these alterations may differ consistently, even if the origins of the tested cell lines were identical. Results converged to the conclusion that CC cells change their morphology and cytotoxicity and react differently to L-OHP therapy by activating genes and upstream regulators that resulted in a more primitive, invasive and migratory, therefore more resistant phenotype. By the identification and description of the cellular and molecular alterations that occurred during the chemoresistance acquiring process the present study aims to contribute to a better understanding of this multifactorial process essential in our attempt to reverse chemoresistance and to identify potential targets for future therapies.

## Results

### Morphology analysis

**Figure 1:**
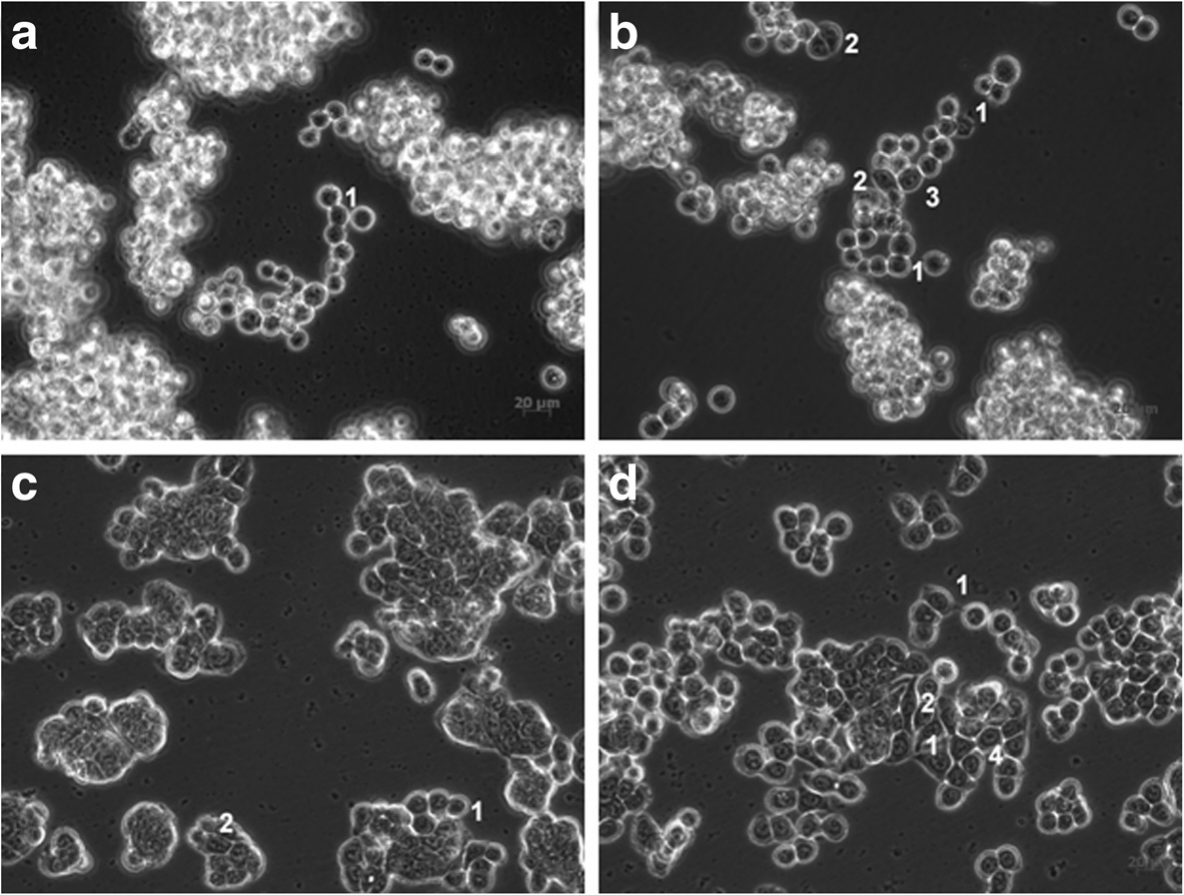
**Microscopic images of Colo320 (a), Colo320R (b), HT-29 (c) and HT-29R (d) CC cell lines.** Morphologic analyses revealed round (a1, c1) and polygonal (c2) types for the sensitive cells. For the resistant ones, transitional (d1) and fusiforme (b2, d2) types of cells were identified and specific alterations for epithelial-to-mesenchymal transition: presence of pseudopodia (b1; d1), loss of cell polarity and adoption of fusiforme shape (b2; d2), increased adherence for the generally non-adherent Colo320R (b3) and increased cell-to-cell distance for the HT-29R cells (d4).

The microscopic analysis revealed distinct morphologic features for the cells with acquired resistance to L-OHP as compared to the parental ones. Some of the Colo320R cells have lost their globular shapes and became fusiforme and adherent, contrasting to the suspension-type parental ones. HT-29R cells displayed loss of cell polarity, the mainly polygonal cells becoming fusiforme, through several oblong transitional forms. An increased cell-to-cell distance between the adherent HT-29R cells was observed and the presence of pseudopodia in both of the resistant cell lines (Colo320R and HT-29R) (Figure 1).

### Cytotoxicity assessment

The cytotoxicity of L-OHP on the selected CC cell lines was calculated using a sigmoid-type non-linear regression method. 2.76 (*P* < 0.0001) and 2.54 (*P* < 0.001)-fold increases of IC_50_ values were recorded in Colo320R and HT-29R cell lines, respectively (Table 1). The significantly higher IC_50_ values obtained for the L-OHP-treated cells vs their parental analogues confirmed the induction of chemoresistance in both of the tested cell lines. We also found that, at the same dose range, Colo320 was almost 3-fold more sensitive to L-OHP than HT-29 cell line (*P* < 0.001).

**Table 1:** Cytotoxicity of L-OHP in parental and their L-OHP resistant variants (Colo320/Colo320R and HT-29/HT-29R)

**Cell lines**	**Colo320**	**Colo320R**	**HT-29**	**HT-29R**
IC_50_ (μg/ml)	7.546 ± 0.5970	20.85 ± 1.069	22.31 ± 2.717	56.80 ± 5.065

Values are means ± SEM in triplicate, in 3 separate experiments, after 24 h exposure of the cells to varying concentrations of L-OHP (0.001-300 μg/ml).

### Evaluation of the DNA cross-links induced by L-OHP

**Figure 2:**
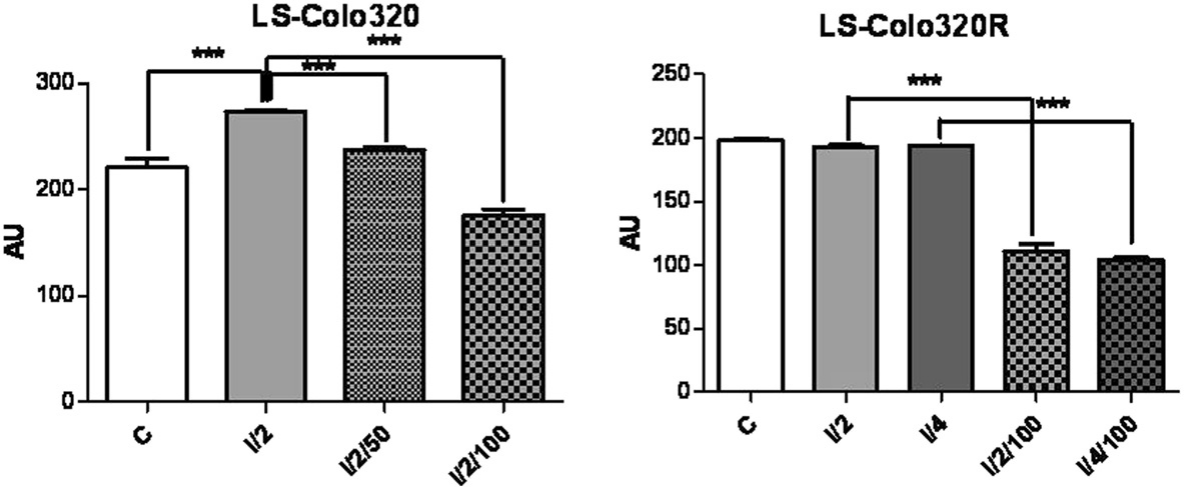
**Representation of lesion scores (LS) of the Colo320 and Colo320R CC cell lines.** Controls (C); irradiated with doses of 2Gy (I/2) and 4Gy (I/4) of gamma irradiations; exposed to 50 μg/ml or 100 μg/ml L-OHP and irradiated with a dose of 2Gy radiations (I/2/50 and I/2/100, respectively); exposed to 100 μg/ml L-OHP and irradiated with doses of 2Gy (I/2/100) and 4Gy (I/4/100); values are means of three experiments (*** p < 0.0001, one-way analysis of variance test).

The lesion scores (LS) calculated in CA revealed that irradiation of the parental cells at a dose of 2 Gy caused important DNA strand-breaks, Colo320 being more radiosensitive than HT-29 (P < 0.0001 vs. 0.001) (Figure 2), i.e. more affected by this genotoxic agent.

The exposure of these cells to different concentrations of L-OHP prior to irradiation reduced significantly the length of the comet tails, due to the formation of L-OHP-DNA cross-links. This reduction was significant and dose-dependent in Colo320 (*P* < 0.0001), for both concentrations of the drug; for HT-29 this reduction was less important (*P* < 0.001 at 50 μg/ml and *P* < 0.05 at 100 μg/ml L-OHP) (Figure 2).

**Figure 3:**
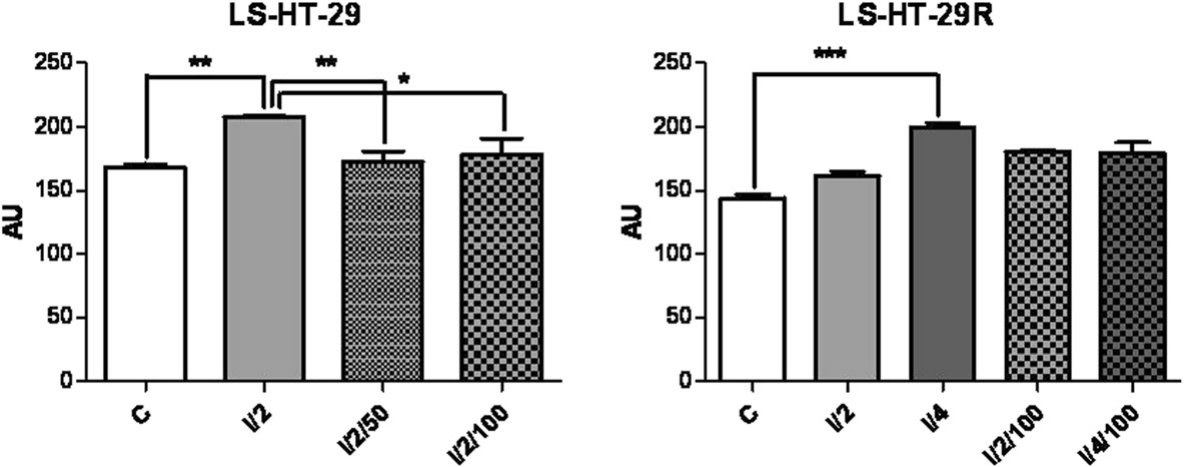
**Representation of lesion scores (LS) of the HT-29 and HT-29R CC cell lines.** Controls (C); irradiated with doses of 2Gy (I/2) and 4Gy (I/4) of gamma irradiations; exposed to 50 μg/ml or 100 μg/ml L-OHP and irradiated with a dose of 2Gy radiations (I/2/50 and I/2/100, respectively); exposed to 100 μg/ml L-OHP and irradiated with doses of 2Gy (I/2/100) and 4Gy (I/4/100); values are means of three experiments (* p < 0.05, ^**^ p < 0.001 and *** p < 0.0001, one-way analysis of variance test).

The L-OHP resistant cells displayed different responses to these treatments, compared to their sensitive counterparts. Irradiation with 2 Gy produced insignificant increases in the LS for both cell lines. Preliminary experiments showed that 50 μg/ml L-OHP did not cause DNA damages in the resistant variants, neither in the drug-treated and irradiated nor in the corresponding drug-treated non-irradiated samples (data not shown). Therefore, we increased the dose of irradiation (4 Gy) and the concentration of L-OHP (100 μg/ml). 4 Gy caused significant DNA lesions in HT-29R cell line (*P* < 0.0001) as compared to control, but these effects were not observable in Colo320R (Figure 3). The administration of L-OHP (100 μg/ml) prior to irradiation did not modify notably the LS in HT-29R, serving as a proof for the acquired drug-resistance. In Colo320R, the administered L-OHP (100 μg/ml) still provoked cross-links (P < 0.0001 for both irradiation doses) (Figure 3).

### Gene expression profiles in the tested cells

**Figure 4:**
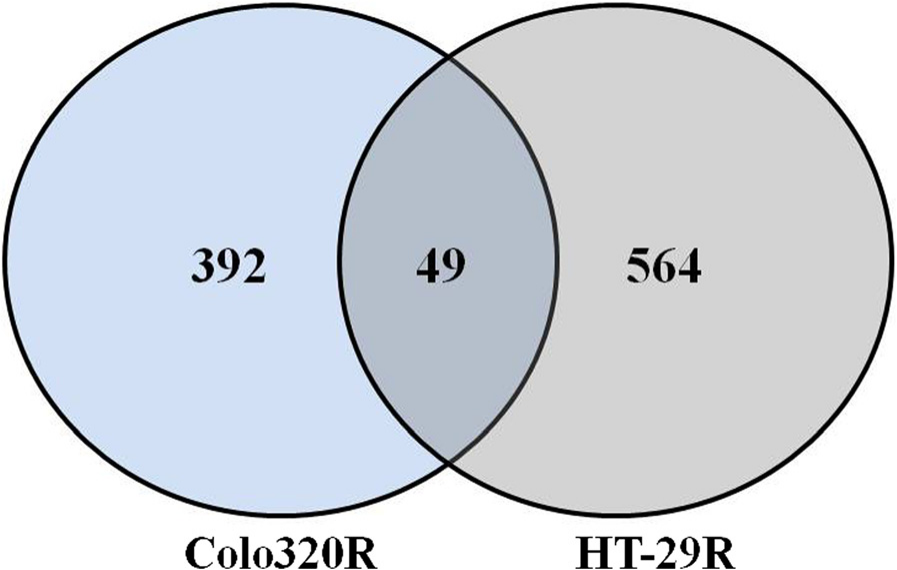
**Venn diagram of DE genes induced by L-OHP in tested cell lines.** The overlap area indicates the common set of genes (sequences) modulated by L-OHP in Colo320R and HT-29R cell lines. In the left area is represented the number of the genes (392) modulated by L-OHP only in Colo320R, whereas in the right area is represented the number of the genes (564) uniquely modulated by the drug in HT-29R cell line.

In order to identify the molecular changes that occurred during the resistance acquiring process in CC cells, we performed class comparison analysis of the parental cell lines (Colo320 and HT-29) and their resistant counterparts (Colo320R and HT-29R). Using an M value cut-off of 0.58 (1.5 fold regulation) and an adjusted p value < 0.05, we found 441 DE genes in Colo320R vs Colo320, representing 1.33% of the analyzed genes. Applying the same criteria of selection, we identified 613 DE genes in HT-29R vs HT-29 (1.85%). Of the total number of DE genes modulated by L-OHP in Colo320R, 260 (59%) were up regulated and 181 (41%) were down regulated. In HT29R we identified 349 (57%) over expressed and 264 (43%) under expressed genes out of 613. As shown in Venn diagram (Figure 4), although the percentages of the DE genes induced by L-OHP in the tested cell lines were comparable our results revealed that 392 genes were modulated exclusively in Colo320R and 564 genes in HT-29R. Only a set of 49 genes (sequences) was identified as commonly modulated by L-OHP in both cell lines. As 13 sequences were uncharacterized, we removed them from further analysis. Finally, we observed that 9 out of the 36 common genes had different profiles in the two cell lines, while 27 genes exhibited similar profiles (Table 2).

**Table 2:** Common core set of DE genes modulated by L-OHP in the tested cell lines

**Gene symbol**	**Gene name**	**Colo 320R**		**HT-29R**	
		**Fold regulation**	**p value**	**Fold regulation**	**p value**
**Class A**					
TMX4	Thioredoxin-related transmembrane protein 4	**3.43**	1.18E-06	**2.73**	6.57E-10
PTPRO	Protein tyrosine phosphatase, receptor type, O	**1.58**	2.58E-05	**1.85**	1.93E-08
LGALS1	Lectin, galactoside-binding, soluble, 12	**2.71**	7.32E-07	**1.58**	3.62E-09
KRT18	Keratin 18, transcript variant 1	**1.87**	7.29E-08	**1.54**	1.81E-07
THC2739159	ALU8_HUMAN (P39195) Alu subfamily SX sequence contamination warning entry, partial (8%)	**2.04**	2.48E-04	**1.77**	7.46E-08
XR_019191	PREDICTED: Homo sapiens similar to Keratin, type I cytoskeletal 18 (Cytokeratin-18) (CK-18) (Keratin-18) (K18) (LOC121054), mRNA [XR_019191]	**2.00**	7.97E-09	**1.56**	6.13E-08
XR_017030	Heat-shock protein beta-1 (HspB1) (Heat shock 27 kDa protein) (HSP 27) (Stress responsive protein 27) (SRP27) (Estrogen-regulated 24 kDa protein) (28 kDa heat shock protein)	**1.99**	1.30E-08	**1.53**	8.82E-08
XR_018843	PREDICTED: Homo sapiens similar to Keratin, type I cytoskeletal 18 (Cytokeratin-18) (CK-18) (Keratin-18) (K18) (LOC649233), mRNA [XR_018843	**1.93**	8.66E-07	**1.71**	1.52E-05
AK022045	Homo sapiens cDNA FLJ11983 fis, clone HEMBB1001337	**1.80**	1.24E-05	**1.82**	6.51E-09
THC2611661	RR12_SPIMX (P42344) Chloroplast 30S ribosomal protein S12, partial (11%)	**1.80**	2.24E-04	**1.51**	8.50E-08
THC2524582	Q5U0N8_HUMAN (Q5U0N8) Keratin 18 (Cell proliferation-inducing protein 46), partial (46%) [THC2524582]	**1.75**	3.60E-07	**1.57**	8.96E-07
NM_173624	KRT18P55 keratin 18 pseudogene 55	**1.72**	3.34E-07	**1.57**	1.13E-06
XR_018670	PREDICTED: Homo sapiens similar to Keratin, type I cytoskeletal 18 (Cytokeratin-18) (CK-18) (Keratin-18) (K18) (LOC643471), mRNA [XR_018670]	**1.72**	1.71E-07	**1.51**	6.88E-08
XR_015605	PREDICTED: similar to Keratin, type I cytoskeletal 18 (Cytokeratin-18) (CK-18) (Keratin-18) (K18) (LOC731794), mRNA	**1.71**	3.34E-07	**1.51**	4.97E-07
BC048264	Homo sapiens hypothetical protein LOC283666, mRNA (cDNA clone IMAGE:4415549), partial cds	**1.71**	3.63E-05	**1.88**	1.27E-08
XR_018311	PREDICTED: Homo sapiens similar to Keratin, type I cytoskeletal 18 (Cytokeratin-18) (CK-18) (Keratin-18) (K18) (LOC139060), mRNA [XR_018311]	**1.67**	2.29E-05	**1.55**	1.23E-07
NM_001007139	Homo sapiens insulin-like growth factor 2 (somatomedin A) (IGF2), transcript variant 2, mRNA	**2.08**	7.10E-06	**1.53**	6.85E-09
THC2663167	ALU1_HUMAN (P39188) Alu subfamily J sequence contamination warning entry, partial (5%) [THC2663167]	**2.07**	3.24E-04	**1.55**	1.17E-08
**Class B**					
IL1RAP	Interleukin 1 receptor accessory protein	**-1.61**	2.86E-05	**-1.53**	4.67E-07
GRB14	Growth factor receptor-bound protein 14	**-1.73**	3.97E-07	**-1.56**	4.16E-09
RBPMS2	RNA binding protein with multiple splicing 2	**-1.60**	1.19E-04	**-1.58**	8.16E-07
RHOBTB1	Rho-related BTB domain containing 1	**-1.58**	7.77E-05	**-1.67**	3.44E-07
CD302	CD302 molecule	**-1.88**	2.57E-06	**-1.68**	1.07E-09
HMGCS1	3-hydroxy-3-methylglutaryl-Coenzyme A synthase 1	**-1.56**	2.38E-04	**-1.73**	2.77E-07
POLR1D	Polymerase (RNA) I polypeptide D, 16 kDa	**-1.52**	1.44E-06	**-1.74**	3.02E-10
CA12	Carbonic anhydrase XII (CA12), transcript variant 1	**-2.60**	2.55E-10	**-1.95**	4.95E-11
AKR1B1	Aldo-keto reductase family 1, member B10	**-1.91**	1.78E-07	**-3.18**	2.54E-12
**Class C**					
IFITM3	Interferon induced transmembrane protein 3	**-1.74**	1.87E-07	**2.08**	1.28E-11
IFITM4P	Interferon induced transmembrane protein 4 pseudogene	**-1.60**	3.88E-07	**1.78**	1.28E-08
IFIH1	Interferon induced with helicase C domain 1	**-2.64**	1.40E-07	**1.67**	4.51E-08
CFHR1	Complement factor H-related 1	**-2.64**	4.32E-09	**1.61**	1.70E-08
**Class D**					
CAMK2N1	Calcium/calmodulin-dependent protein kinase II inhibitor 1	**1.64**	1.33E-07	**-1.55**	3.97E-07
SAMD5	Sterile alpha motif domain containing 5	**1.51**	2.41E-04	**-1.73**	8.09E-09
NDRG1	N-myc downstream regulated 1	**1.79**	7.96E-07	**-1.75**	1.55E-07
COL9A3	Collagen, type IX, alpha 3	**1.91**	3.92E-06	**-1.79**	6.45E-09
IRX5	Iroquois homeobox 5	**1.88**	1.94E-07	**-1.53**	9.56E-08

Classes A and B represent up- respectively down-regulated genes induced by L-OHP in Colo320R and HT-29R. Classes C and D include antagonist DE genes induced by L-OHP in the tested cell lines.

### Identification of the biological pathways modulated by L-OHP

In order to assess the molecular functions and canonical pathways [12] modulated by L-OHP in the tested cell lines we performed the IPA Core Analysis. 334 out of 441 DE genes in Colo320R and 492 out of 612 DE genes in HT-29R were mapped in IKB. The most significant cellular and molecular functions affected by L-OHP in Colo320R and HT-29R cell lines were related to cell death and survival, cellular growth and proliferation, DNA replication, cellular movement and cell-to-cell signaling. The analysis of the canonical pathways revealed 15 and 23 canonical pathways significantly modulated by L-OHP in Colo320R and HT-29R (p < 0.05) cell lines, respectively (Tables 3, 4).

**Table 3:** Top canonical pathways modulated by L-OHP in Colo320R

**Canonical pathways Colo320R**	**p-value**	**Log Ratio**	**Molecules**
**Aryl hydrocarbon receptor signaling**	1E-03	5.59E-02	HSPB3, CDKN2A, MYC, FOS, GSTM2, ALDH1A1, NQO1, TGFB2, HSPB1
**HGF Signaling**	3E-03	6.67E-02	CDKN2A, MET, FOS, MAP3K6, MAP3K13, ETS2, ELK3
**LPS/IL-1 mediated inhibition of RXR function**	7E-03	4.18E-02	CHST2, ABCB1, GSTM2, ALDH1A1, SLC27A2, HS3ST1, CPT1C, PLTP, HMGCS1, IL1RAP
**Hepatic fibrosis/hepatic stellate cell activation**	1.6E-02	4.79E-02	MET, TIMP1, ACTA2, TGFB2, IGFBP5, IL1RAP, COL3A1
**Retinoate biosynthesis I**	1.8E-02	7.89E-02	ALDH1A1, RBP7, AKR1C3
**Methylglyoxal degradation III**	1.68E-02	8.7E-02	AKR1C3, AKR1B1
**S-methyl-5′-thioadenosine degradation II**	1.9E-02	1.67E-01	MTAP
**Histamine biosynthesis**	1.9E-02	3.33E-01	HDC
**Retinol biosynthesis**	2.5E-02	4.92E-02	DHRS3, RBP7, LPL
**Cell Cycle: G1/S checkpoint regulation**	2.7E-02	6.06E-02	CDKN2A, MYC, HDAC9, TGFB2
**The visual cycle**	2.9E-02	7.14E-02	DHRS3, RBP7
**PXR/RXR activation**	3.2E-02	4.6E-02	ABCB1, GSTM2, ALDH1A1, FOXO1
**Glutamine biosynthesis I**	3.8E-02	1.25E-01	GLUL
**Thyroid cancer signaling**	4.2E-02	7.14E-02	PPARG, MYC, NTF3
**Mitochondrial L-carnitine shuttle pathway**	4.2E-02	9.09E-02	SLC27A2, CPT1C

**Table 4:** Top canonical pathways modulated by L-OHP in HT-29R

**Canonical pathways HT-29R**	**p-value**	**Log ratio**	**Molecules**
**Interferon signaling**	2,51E-05	1.94E-01	IFIT3, IFIT1, IFITM1, MX1, IRF9, PSMB8, STAT1
**Methylglyoxal degradation III**	2,04E-04	1.74E-01	AKR7A3, AKR1C1/AKR1C2, AKR1B10, AKR1B1
**Cholesterol biosynthesis I**	4,17E-04	1E-01	SQLE, DHCR7, MSMO1, CYP51A1
**Cholesterol biosynthesis II (via 24,25-dihydrolanosterol)**	4,17E-04	1E-01	SQLE, DHCR7, MSMO1, CYP51A1
**Cholesterol biosynthesis III (via Desmosterol)**	4,17E-04	1E-01	SQLE, DHCR7, MSMO1, CYP51A1
**LXR/RXR activation**	7,76E-04	8.09E-02	SCD, TTR, LDLR, SREBF1, AMBP, SERPINA1, PTGS2, TLR3, IL1RAP, CYP51A1, AGT
**Mevalonate pathway I**	4,47E-03	1.07E-01	IDI1, HMGCS2, HMGCS1
**Creatine-phosphate biosynthesis**	4,90E-03	3.33E-01	CKB, CKMT1A/CKMT1B, LCMT2
**Activation of IRF by Cytosolic pattern recognition receptors**	5,75E-03	8.33E-02	DHX58, IFIH1, IRF9, STAT1, IFIT2, ISG15
**Tryptophan degradation X (Mammalian, via Tryptamine)**	1,05E-02	1.03E-01	ALDH1A3, DDC, MAOA
**Zymosterol biosynthesis**	1,17E-02	9.09E-02	MSMO1, CYP51A1
**Maturity onset diabetes of young (MODY) signaling**	1,95E-02	9.68E-02	FOXA2, FABP1, HNF4A
**Eicosanoid signaling**	2,19E-02	6.25E-02	DPEP1, PNPLA3, RARRES3, PTGS2, ALOX5
**Ketogenesis**	2,69E-02	9.52E-02	HMGCS2, HMGCS1
**Acyl-CoA hydrolysis**	3,31E-02	1.25E-01	ACOT4, HNF4A
**PXR/RXR activation**	3,47E-02	5.75E-02	SCD, NR0B2, CYP2B6, HMGCS2, HNF4A
**Colorectal cancer metastasis signaling**	3,89E-02	4.67E-02	VEGFA, MMP7, RND3, TGFB1, FZD3, GNB5, TGFB2, PTGS2, TLR3, STAT1, FNBP1, WNT5A
**Aryl hydrocarbon receptor signaling**	4,17E-02	4.97E-02	TGM2, TFF1, NR0B2, ALDH1A3, TGFB1, TGFB2, ALDH18A1,NRIP1
**p38 MAPK signaling**	4,27E-02	5.98E-02	DDIT3, TGFB1, TGFB2, MKNK2, RPS6KA2, STAT1, IL1RAP
**Choline biosynthesis III**	4.64E-02	9.09E-02	CHPT1,PLD1
**Histamine degradation**	4.64E-02	6.9E-02	ALDH1A3,ABP1
**Adenosine nucleotides degradation II**	4.64E-02	8E-02	NT5E, ADA
**Wnt/β-catenin signaling**	5E-02	5.2E-02	MMP7, CDH3, TGFB1, PPP2R3A, TGFBR3, FZD3, CD44, TGFB2, WNT5A

### Identification of upstream regulators induced by L-OHP

**Figure 5:**
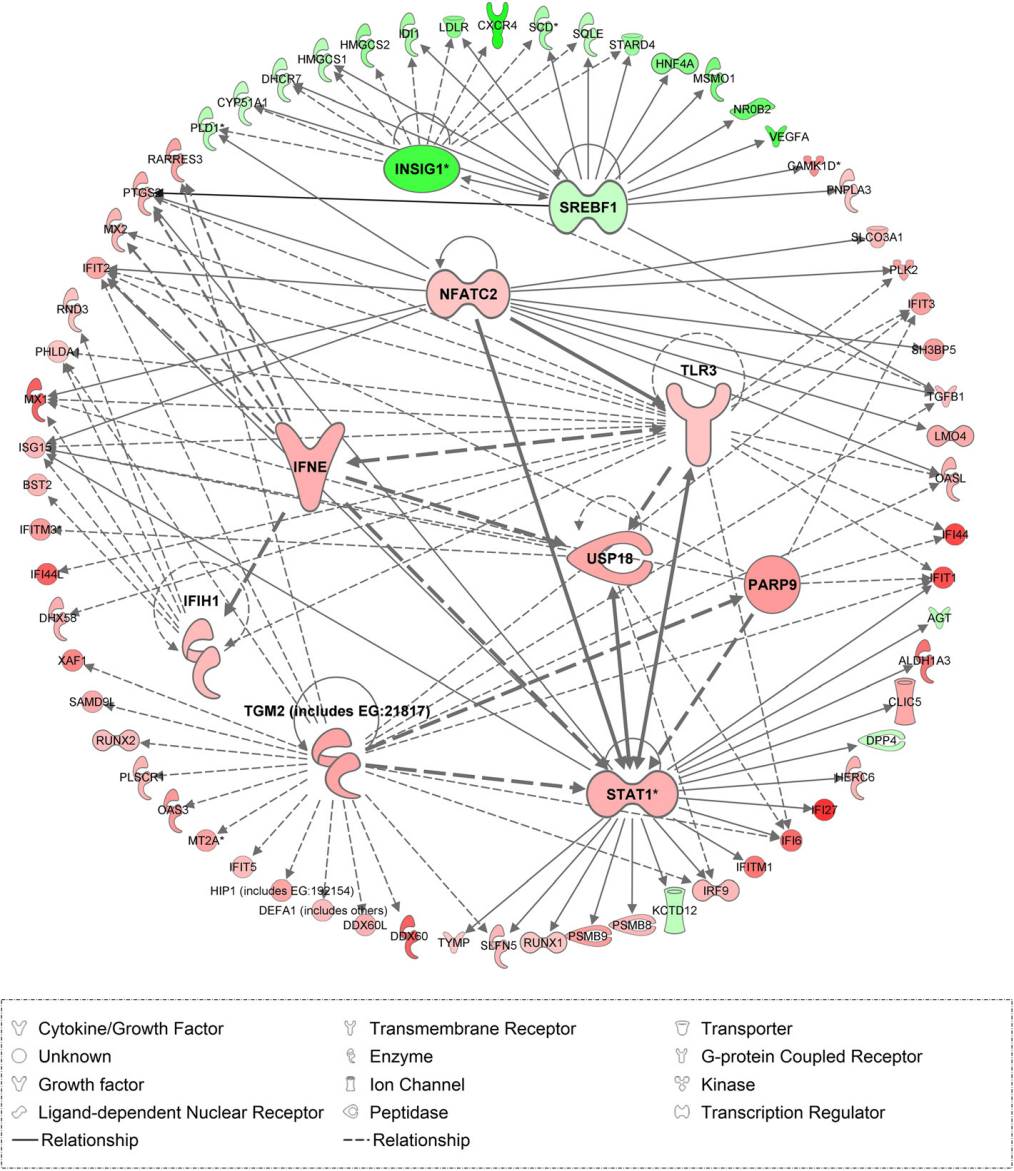
**IPA Network.** The network displays the relationship between upstream regulators and their target molecules in HT-29R cell line. The colors indicate the level of mRNA expression: upregulated genes are represented in red and downregulated genes in green.

Using IPA Upstream Regulator Analysis, we identified 10 upstream regulators and their target molecules in HT-29R dataset. Eight modulators were predicted to be “activated” (z score > 2, p < 0.01) while 2 modulators were predicted as “inhibited” (z score < 2, p < 0.01) in response to L-OHP treatment. These regulators were involved in transcription, enzyme activity and signal transduction (Table 5). We also used IPA network overlay function to investigate the relationships between these upstream regulators and their target genes (Figure 5). Conversely, we didn’t find significant upstream regulators in Colo320R dataset.

**Table 5:** Upstream regulators induced by L-OHP treatment in HT-29R

**Upstream regulator**	**Log ratio**	**Molecule type**	**Predicted activation state**	**z-score**	**p-value of overlap**	**Target molecules in dataset**
TGM2	0.968	Enzyme	Activated	4.762	8.90E-09	DDX60, DDX60L, DEFA1, HIP1, IFI6, IFIT1, IFIT2, IFIT3, IFIT5, IRF9, MT2A, OAS3, OASL, PARP9, PHLDA1, PLSCR1, RARRES3, RUNX2, SAMD9L, SLFN5, STAT1, TGFB1, XAF1
STAT1	0.855	Transcription regulator	Activated	3.592	1.47E-08	AGT, ALDH1A3,CLIC5, DPP4, HERC6, IFI27, IFI6, IFIT1, IFIT2, IFITM1, IRF9, ISG15, KCTD12, PSMB8, PSMB9, PTGS2, RUNX1, SLFN5, STAT1, TLR3, TYMP, USP18
TLR3	0.624	Transmembrane receptor	Activated	3.392	5.85E-07	DHX58, IFI44, IFI44L, IFI6, IFIH1, IFIT1,IFIT2, IFIT3, IFNE, ISG15, MX1, MX2, OASL, PHLDA1, PLK2, PTGS2, STAT1, TLR3, USP18
INSIG1	-2.061	Other	Activated	3.039	1.74E-06	CXCR4, CYP51A1, DHCR7, HMGCS1, HMGCS2, IDI1, LDLR, PLD1, SCD, SQLE, SREBF1, STARD4,TGFB1
NFATC2	0.635	Transcription regulator	Activated	3.019	3.84E-05	IFIT2, ISG15, LMO4, MX1, OASL, PLD1, PLK2, PTGS2, SH3BP5, SLCO3A1, STAT1, TGFB1,T LR3
IFNE	0.861	Cytokine	Activated	2.804	2.07E-07	IFIH1, IFIT2, MX2, PTGS2, RARRES3, STAT1, TLR3, USP18
PARP9	1.087	Other	Activated	2.433	1.24E-06	IFI44, IFIT1, IFIT2, IFIT3, ISG15, STAT1
IFIH1	0.742	Enzyme	Activated	2.190	2.68E-04	BST2, ISG15, MX1, PHLDA1, RND3
SREBF1	-0.624	Transcription regulator	Inhibited	-2.980	1.36E-07	CAMK1D, CYP51A1, DHCR7, HMGCS1, HNF4A, IDI1, INSIG1, LDLR, MSMO1, NR0B2, PNPLA3, PTGS2, SCD, SQLE, SREBF1, STARD4,T GFB1, VEGFA
USP18	0.941	Peptidase	Inhibited	-2.219	5.62E-05	IFI6, IFITM3, IRF9, ISG15, MX1

### Validation of microarray results by qRT-PCR

**Figure 6:**
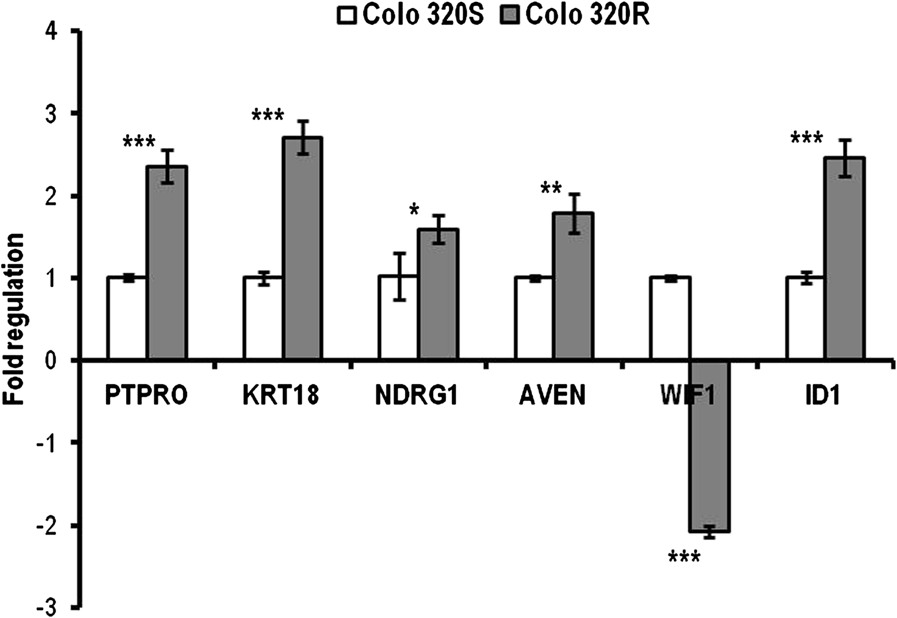
**qRT-PCR validation of microarray results in Colo320 cell line.** The bars represent the mean (± SD) of three biological replicates for every gene. All genes were normalized to 18 rRNA and fold regulation was calculated relative to Colo320S. (* p < 0.05, ** p < 0.01, *** p < 0.001).

**Figure 7:**
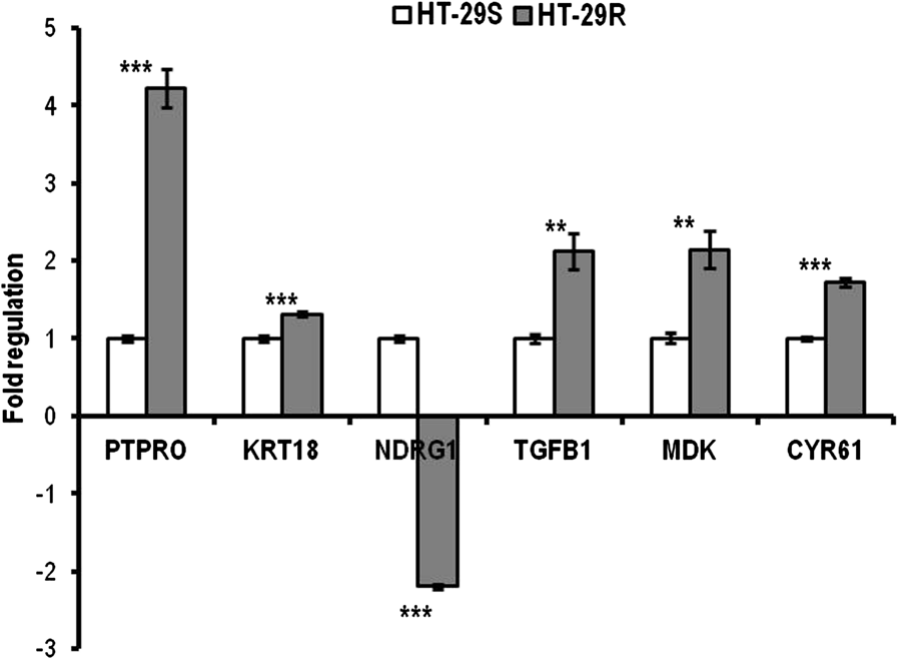
**qRT-PCR validation of microarray results in HT-29 cell line.** The bars represent the mean (± SD) of three biological replicates for every gene. All genes were normalized to 18 rRNA and fold regulation was calculated relative to HT-29S. (* p < 0.05, ** p < 0.01, *** p < 0.001).

In order to assess the reliability of microarray results, we considered 9 DE genes as candidates for validation by qRT-PCR as following: 3 common genes modulated in both cell lines (PTPRO, KRT18, NDRG1), 3 genes modulated exclusively in Colo320R (ID1, WIF1 and AVEN) and 3 in HT29R (TGFB1, MDK, and CYR61) cell lines, respectively (Figures 6, 7). The qRT-PCR results were consistent with the microarray data. A significant and strong correlation was found between the microarray and qRT-PCR data for both Colo320 (r = 0.97, p = 0.0009) and HT29 (r = 0.89, p = 0.016) cell lines (Table 6).

**Table 6:** The correlation between microarray and qRT-PCR results

**Accession #**	**Gene symbol**	**Gene name**	**Microarray**		**qRT-PCR**		**Pearson correlation r (p)**
			**Fold regulation**	**p value**	**Fold regulation**	**p value**	
**Colo320R**							
NM_030667	PTPRO	Protein tyrosine phosphatase, receptor type, O	1.58	2.58E-05	2.36	3E-04	0.97 (9E-04)
NM_000224	KRT18	Keratin 18, transcript variant 1	1.87	7.29E-08	2.71	2E-04	
NM_006096	NDRG1	N-myc downstream regulated 1	1.78	7.96E-07	1.55	0.04	
NM_020371	AVEN	Apoptosis, caspase activation inhibitor	1.50	4.74E-06	1.79	5E-03	
NM_007191	WIF1	WNT inhibitory factor 1	-1.72	2.35E-07	-2.08	3E-04	
NM_002165	ID1	Inhibitor of DNA binding 1, dominant negative helix-loop-helix protein	2.20	1.97E-08	2.45	4E-04	
**HT-29R**							
NM_030667	PTPRO	Protein tyrosine phosphatase, receptor type, O	1.85	1.93E-08	4.22	2.70E-05	0.89 (1.6E-02)
NM_000224	KRT18	Keratin 18, transcript variant 1	1.54	1.81E-07	1.31	4E-04	
NM_006096	NDRG1	N-myc downstream regulated 1	-1.75	1.55E-07	-2.17	4E-04	
NM_000660	TGFB1	Transforming growth factor, beta 1	1.66	1.10E-09	2.12	0.001	
NM_001012334	MDK	Midkine (neurite growth-promoting factor 2)	2.22	3.51E-11	4.29	0.001	
NM_001554	CYR61	Cysteine-rich, angiogenic inducer, 61	1.56	5.62E-09	1.72	4.49E-05	

## Discussion

Oxaliplatin has antitumoral activity against colorectal, breast, gastric, renal carcinomas and sarcomas [13] mainly by forming intrastrand cross-links that block DNA replication and transcription. Despite the improvements in the treatment outcome for CC, the tumor cells acquire resistance in time, decreasing the drug’s clinical efficiency.

To address this issue we assessed the morphology, cytotoxicity, DNA cross-links induction and gene expression profiles of two colorectal cell lines with identical origins (adenocarcinoma) with acquired resistance to L-OHP and their parental lines. According to our results the L-OHP resistant cells displayed altered cellular and molecular features as compared to the parental cells. In addition, notable differences were recorded between the functions and pathways modulated by L-OHP in the two tested cell lines.

Some of the morphological alterations we observed here: pseudopodia formation and adoption of fusiforme shape, suggesting an epithelial-to-mesenchymal transition (EMT) and an increased cell-to-cell distance in the HT-29R cells were also identified by Yang et al. in chemoresistant HT-29 cells [14]. Part of the embryonic development, EMT appears to be involved in tumor progression and metastasis [15,16], a process through which cells switch from the proliferative state to a more primitive, invasive and migratory mode. This conversion was proposed as a potential mechanism through which cells become chemoresistant, being known that the cytostatic drugs are more efficient on the highly proliferative cells [14]. In our study Colo320R cells displayed (a mesenchymal phenotype, but adopted some) different characteristics after prolonged treatment with L-OHP. These cells, usually exposed in suspension, reacted to the prolonged treatment with the cytostatic drug by showing an increased tendency of adherence. Although our findings demonstrated different adaptations of the tested cell lines to the L-OHP treatment, a common feature was obvious: the alteration of the cellular adhesion complexes, suggesting higher invasiveness and attachment capacity.

The IC_50_ values obtained in the present study revealed that the prolonged treatment of the cells with increasing concentrations of the drug, up to the clinically relevant concentration (2 μmol/l), induced resistance in the treated cells as compared to the parental ones. Our results are comparable to other previous findings on parental and resistant Colo320 cells [11] and on sensitive Colo320 and HT-29 cells [7,17].

The CA findings confirmed different behaviors of the tested cell lines to the prolonged treatment to L-OHP. Both parental cell lines were sensitive to 2 Gy of gamma irradiation and displayed consistent DNA damages. The administration of L-OHP prior to irradiation revealed higher cross-links formation in the Colo320 cell line as compared to HT-29. These results are in agreement with the cytotoxicity findings which suggested that Colo320 cell line is more sensitive to L-OHP than HT-29. An intriguing fact was the lack of response to the same dose of ionizing radiation (2 Gy) in the chemoresistant cell groups. Moreover, the higher dose of irradiation (4 Gy) caused DNA lesions only in HT-29R cells, while Colo320R remained unresponsive. These results suggest that acquired resistance to a chemotherapeutic agent could have activated general resistance pathways that impart resistance to multiple agents, including irradiation. Another potential explanation for the lack of the response to irradiation could be the presence of some free radical scavengers that might have contributed to the resistance to irradiation. The redox homeostasis of the cells was previously implicated in chemo-resistance. It is commonly accepted that the sensitivity of tumor cells to L-OHP, can be influenced by gluthatione and gluthatione related enzymes [18]. Thus, gluthatione S-tranferase (GST) catalyses the conjugation of gluthatione to genotoxic compounds, preventing DNA-damage and adduct formation [19]. Gluthatione system limits the cytotoxic activity of oxaliplatin by modifying the production of cellular reactive species (ROS) [20]. If ROS production decreases, also the cytotoxicity of the chemotherapy agent decreases. Additionally, ROS act also as critical mediators in ionizing radiation-induced cell killing [21]. Moreover, some subsets of tumor cells were shown to possess lower levels of ROS and enhanced ROS defense, which contributed to their radioresistance [22]. Therefore, a link between redox homeostasis and cells’ resistance to chemo/radiotherapy is conceivable. Given that this subset of cells, with increased radioresistance was ascertained as cancer stem cells (CSC), we can presume that another cause of the cells’ peculiar response to ionizing irradiation could be the phenotypic characteristics of the cells. Resistant cells in our study have switched their morphology, as our microscopy findings suggest it. It is also possible that this population might have adopted stem-like characters. Literature data sustain that cancer stem cells (CSC), expressing CD133+ marker manifest resistance to irradiation with 2, 5 Gy unlike CD133- non stem-cells [23]. This different behavior was explained by the activation of the DNA damage checkpoint more efficiently in CSC than in tumor cells without stem cells properties, due to the activation of Chk1 and Chk2 checkpoint kinases [24]. We also demonstrated in the present study the phosphorylation of Chk2 and p53, due to activation of ataxia-telangiectasia mutated gene ATM, this being activated by an apoptosis caspase activation inhibitor (AVEN). Activation of AVEN being evident in Colo320R cell line and not in HT-29R may be a possible explanation for the differential behavior of these cells as compared to HT-29R (i.e. HT-29R responded to 4 Gy, unlike Colo320, which remained resistant). The significantly different gene expression profiles of the tested cell lines, sustained also by cytotoxicity suggest that these cells have completely different genomic patterns, which may explain also their different behavior towards gamma irradiation. The fact that in Colo320R cell line we still could detect cross-links confirms once more the higher sensitivity of this cell line to L-OHP as compared to HT-29. In a previous study we observed changes in the comet tails length according to the degree of the treatment with L-OHP, indicating the presence or absence of the DNA cross-links induced by L-OHP on the tested cell lines [11]. In another study we induced standardized DNA strand breaks via ionizing irradiation and compared the cross-links formation capacity of L-OHP vs CDDP in 3 CC cell lines (Colo320, Caco-2 and HT-29) [17]. Our results showed higher cytotoxicity and cross-links formation for L-OHP vs CDDP in the tested cell lines, in spite of lower cellular uptake. In the present study, we determined indirectly the cross-links formation on the irradiation-damaged DNA and demonstrated that L-OHP resistant cells form fewer cross-links than the parental ones, in accordance with the sensitivity of the tested cells to the cytotoxic drug. Similar studies were performed by others treating with CDDP the healthy human leukocytes [25] and ovarian carcinoma cells [26] and using L-OHP treatment on lymphocytes and lung carcinoma cell lines [27]. These studies used UV-C, methyl methanesulfonate and ionizing radiations, respectively, as DNA strand-breaking agents and detected DNA cross-links using an *in vitro* measure of the cells’ chemosensitivity to the tested compounds.

Our microarray data were in agreement with the morphology, cytotxicity and DNA lesions findings showing that the prolonged treatment with L-OHP triggered different patterns in the transcriptional profiles of the two tested cell lines. To our knowledge, there are no similar studies to highlight the differences between the molecular patterns of these two resistant cell lines however there are genomics studies that evaluated the resistance to treatment either in Colo320 or HT-29 [28]. Considering the common origin of these cell lines (adenocarcinomas) and the mechanism of action of L-OHP which blocks DNA replication and transcription through the formation of intra-strand DNA adducts, we would expect at least to some extent, similar molecular and cellular behavior. Surprisingly, our microarray data have revealed only a common core set of 36 genes modulated more than 1.5-fold in both cell lines (p < 0.05) of which just 27 genes exhibited similar profiles (Table 2). These results could be partly explained by the distinct morphology (suspension vs. adherent) and by the intrinsic differences of the two cell lines which emphasize the complexity of the processes that control the resistance acquirement to this cytostatic drug. Our data revealed that L-OHP modulates genes involved in the regulation of some critical mechanisms including DNA replication, cell death and survival, cellular growth and proliferation, cellular movement and cell-to-cell signaling and interaction.

The microarray analysis showed upregulation of keratin 18 (KRT18) and protein tyrosine phosphatase receptor type O (PTPRO), both being involved in apoptosis. The microarray results validated by qRT-PCR confirmed a significant overexpression of these genes in both HT-29R and Colo320R (Table 6). KRT18 was previously identified as being upregulated in colon carcinoma cells [29]. Increased KRT18 expression has been reported to inhibit cytokine-induced death of cervical cancer cells [30] but there are no evidences about the role of KRT18 in L-OHP-induced resistance in CC. PTPRO is a member of family of receptor-type protein tyrosine phosphatases with multiple tissue-specific functions including inhibition of cell proliferation and promoting of apoptosis. PTPRO was identified as a target gene of Wnt/β-catenin signaling [31] and a novel regulator of ERBB2 signaling for mammary epithelial transformation [32]. Ramaswamy et al. observed increased expression of PTPRO in breast cancer following the treatment with tamoxifen [33]. In CC there are no studies describing the implication of PTPRO in drug resistance, but this gene was found to be methylated in colon tumors [34].

The core set of common DE genes also included some members of interferon - inducible transmembrane gene (IFTIM), whose transmembrane proteins are involved in the homotypic cell adhesion functions of interferon (IFN) [35]. We identified significant upregulation of IFITM3, IFITM4P and IFIH1 in HT29R and downregulation of these genes in Colo320R (Table 2, Class C). The overexpression of IFTIM3 is related to an increased proliferation and metastasis of human colon cancer cells. Andreu et al. identified high endogenous levels of IFITM3 in HT29 cells with APC mutated gene [36]. The authors demonstrated that induction of wild-type APC causes a reduction on IFTIM3 genes within 24 hours. In another study, Ghaleb et al. demonstrated that IFITM3 transcription is dependent on activation of Wnt/β-catenin signaling, in intestinal epithelium [37]. This study appears to be in concordance with our results. Analyzing the canonical pathways for both cell lines we noticed an increased activity for Wnt/β-catenin signaling in HT29R but not in Colo320R (Tables 3, 4). These findings support the morphological observations which suggest an epithelial-to-mesenchymal transition in HT-29R cells.

N-myc downstream regulated 1 (NDRG1) gene had a conflicting expression in the two cell lines, being overexpressed in Colo320R and underexpressed in HT-29R (Table 2, Class D). qRT-PCR confirmed upregulation of NDRG1 in Colo320R and downregulation in HT-29R as a result of prolonged treatment with L-OHP (Table 6). The protein encoded by NDRG1 is implicated in p53-mediated caspase activation and apoptosis. Strzelczyk et al. showed correlation between low levels of NDRG1 gene expression and poor prognosis and survival for patients with CC [38]. These results could suggest that lower level of NDRG1 in HT29R than in Colo320R could be related to a more resistant phenotype.

In response to treatment with cytostatic drugs, cells undergo apoptosis according to the drug-induced DNA damage and the cells’ capacity of DNA repair and survival. In Colo320R, the apoptotic process was mediated by genes involved in caspase modulation and cell cycle regulation. Our results showed that apoptosis caspase activation inhibitor (AVEN), Galectin-3 (LGALS3) and nucleolar protein 3 (NOL3) were overexpressed in this cell line. AVEN represents an activator for ataxia-telangiectasia mutated gene (ATM) which has an important role in the repair of DNA breaks [39]. Cell-cycle arrest induced by DNA damage depends on activation of ATM protein kinase, which phosphorylates cell-cycle effectors such as CHEK2 and p53 in order to inhibit cell-cycle progression. LGALS3 and NOL3 are known as downregulators of the enzyme activities of caspase 2, caspase 8 and tumor protein p53. LGALS3 is involved in the resistance of human colon cancers by blocking the death-inducing signaling complex (DISC) formation and recruitment of the apoptosis-initiating protease, procaspase-8 [40]. Conversely, the increased expression of NOL3 reduced the TRAIL-induced apoptosis in SW480 CC cells [41]. We observed an inhibition of cyclin-dependent kinase inhibitor 2A (CDKN2A) and WNT inhibitory factor 1 (WIF1) tumor suppressor genes in Colo320R following the L-OHP treatment. The lack of function of these genes was associated with tumor cell progression [42,43].

In addition to the inhibition of apoptosis, our results pointed out to the activation of the mechanisms involved in promoting cell survival and tumor progression in Colo320R. We observed overexpression of insulin-like growth factor 2 (IGF2), mitogen-activated protein kinase kinase kinase 6 (MAP3K6), FBJ murine osteosarcoma viral oncogene homolog (FOS), inhibitor of DNA binding 1 genes (ID1), involved especially in signal transduction on MAP kinase cascade. Our data are in agreement with the literature data concerning the role and implication of these molecules in CC [44-46]. We also noticed downregulation of the peroxisome proliferator-activated receptor γ (PPARG) in Colo320R. Inhibition of PPARG promotes the cell proliferation and leads to the expression of c-myc and cyclin D1 genes as well as of the beta-catenin protein in the colon epithelium [47].

As we underlined above, the genes evidenced with microarray analysis were quite different between the two tested cell lines, therefore we expected apoptosis in HT-29R to be modulated by a different set of genes compared to those identified for Colo320R. The ineffective induction of apoptosis in HT-29R cell line was mediated by genes involved in Bcl-2 modulation. One of these genes, serum/glucocorticoid regulated kinase 1 (SGK1) is involved in phosphorylation and inactivation of the apoptotic transcription factor forkhead box O3 (FKHRL1), that upregulates death receptor components such as tumor necrosis factor receptor superfamily, member 10 b (TNFRSF10B) and proapoptotic Bcl-2 proteins such as Pim [48]. Another member of Bcl-2 family upregulated in HT-29R cell line, XIAP associated factor 1 (XAF1), has an important role in modulation of apoptosis in tumor cells by inhibiting the caspase-3 activity [49].

We also noticed that the L-OHP resistance in HT-29R was promoted by the overexpression of some important modulators involved in cell proliferation such as: midkine (MDK), cysteine-rich, angiogenic inducer 61 (CYR61), proliferating cell nuclear antigen (PCNA), transforming growth factor beta 1 (TGFB1), spleen tyrosine kinase (SYK) and prostaglandin-endoperoxide synthase 2 (PTGS2). Upregulation of MDK was correlated with tumor progression in oral squamous cell carcinoma [50], but to our knowledge, MDK was not found to be expressed in CC. CYR61 has multiple roles in tumor growth, adhesion and migration, its role as positive growth-regulator in CC being previously described [51]. PTGS2 and PCNA represent two important molecules for the progression of CC and treatment strategy [52] and increased levels of PTGS2 were associated with enhanced tumor cell proliferation and tumorigenesis [53].

Subsequently, we paid attention to upstream regulators in attempt to explain the different phenotypes induced by L-OHP in the two tested CC cell lines. Although statistically significant results were not found in Colo320R, we identified 10 upstream regulators in HT29R, eight out of them with “activated” and two with “inhibited” predictions (Table 5). Among the top predicted regulators in HT29R dataset, the signal transducer and activator of transcription 1 (STAT1) appears to be significantly activated (z-score = 3.592) by L-OHP treatment, its targets being highly enriched in the data (p = 1.47E-08). STAT1 represents an important activator of transcription in CC [54]. The activation of STAT1 in HT-29R was associated with an increased transcriptional activity in a large number of associated interferon-inducible genes (IFITM1, IFIT3, IFIT1, IFI6, IFI27) (Figure 5). We observed that STAT1 is in turn activated by IFNE, NFATC2 and TGM2. The upregulation of STAT1 mediated by interferon epsilon (IFNE) was described on cervical cancer cells [55], but to our knowledge, there are no studies describing the role of IFNE in CC. Nuclear factor of activated T cells (NFATC2) is a transcription factor with an important role in the transcriptional regulation of the immune response. Its role in CC in promoting carcinoma migration and invasion was previously demonstrated in *in vitro* and *in vivo* studies [56,57]. Our data confirm the implication of NAFTC2 in HT-29R CC cell line as an important upstream regulator (z-score = 3.019, p = 3.84E-05). Transglutaminase 2 (TGM2) is an enzyme involved in cell proliferation, differentiation and apoptosis and could mediate chemoresistance in cancer cells [58]. TGM2 was proposed by Miyoshi et al. as a predictive marker for prognosis and therapeutic target in CC [59]. In our study, TGM2 was the most notably activated upstream regulator identified in HT29R cell lines (z-score = 4.762, p = 8.9E-09) (Table 5). Recent studies have shown that TGM2 promotes drug resistance and invasion by inducing a stem cell-like phenotype in ovarian and breast cancer [60,61]. Our results showed that overexpression of TGM2 and NAFTC2 induced TGFB1 activation. High level of TGFB1 expression in HT-29R obtained by microarray was confirmed by qRT-PCR (Table 6). TGFB1 is a well-characterized inducer of EMT in ovarian cancer and human squamous cell carcinoma cells, resulting in increased cell migration and invasion [62,63]. Considering all these findings, upregulation of TGM2, NAFTC2 and TGFB1 in HT29R but not in Colo320R could explain the induction of EMT and acquiring a more resistant phenotype in HT29R.

Another target of TGM2, poly (ADP-ribose) polymerase family member 9 (PARP9) upstream modulator was significantly activated (z-score = 2.433, p = 1.24E-06) in HT-29R and acts as regulator of STAT1. PARP9 was identified as overexpressed in chemoresistant, diffuse large B-cell lymphomas (DLBCLs) [64], but there is no data concerning the implication of PARP9 in CC.

## Conclusions

In our study CC cells adopted several cellular and molecular alterations during the prolonged treatment with L-OHP which led to resistance to this drug. L-OHP resistant cells displayed altered morphologies, higher invasiveness and metastatic capacities, lower cytotoxicities, formed fewer Pt-DNA cross-links and had different gene expression profiles as compared to the sensitive ones. More disrupted functions and pathways were identified in HT-29R than in Colo320R cells, involving genes responsible for apoptosis inhibition, cellular proliferation and epithelial-to-mesenchymal transition. These findings, in agreement with the morphological and cytotoxicity results and the main upstream regulators identified for HT-29R, but not for Colo320R could explain the more resistant phenotype in HT-29R than in Colo320R cell line.

Therefore, we can conclude that prolonged therapy with L-OHP induces different cellular and molecular chemoresistance patterns in CC cells of identical origins (adenocarcinomas). The set of genes modulated by L-OHP and the upstream regulators revealed in our study explain the diverse behavior of the cancer cells to prolonged therapy with L-OHP, moreover could help us to identify some potential means to reverse chemoresistance and consequently to improve the outcome of therapy in CC.

## Methods

### Cell lines and cultures

Colo320 and HT-29 human CC cell lines were obtained from the European Collection of Cell Cultures (ECACC). Colo320 was cultured in RPMI-1640 and HT-29 in McCoy’s 5A Modified Medium, both supplemented with Fetal Calf Serum 10%, L-glutamin and penicillin-streptomycin (Sigma-Aldrich, St. Louis, MO). Experiments were done at 70–80% cell confluence and confirmed in at least three independent experiments.

### Development of L-OHP-resistant cell lines

Resistance to L-OHP (Actavis, Bucharest, Romania) was induced by exposing the cells to increasing concentrations of the drug. The initial dose was 0.01 μg/ml and the final concentration (0.87 μg/ml) corresponded to the clinically relevant plasma concentration of L-OHP (2 μmol/l) [14]. The resistant variant of Colo320 (Colo320R) was obtained and described previously [11]. For the HT-29 cell line we used the same procedure, sequentially increasing concentrations of the drug (with 0.05 μg/ml) being added to the cell culture at every second passage. The surviving cells were grown and propagated every 4–5 days. For both cell lines two groups were considered for investigations: parental (Colo320 and HT-29) and cells with induced chemoresistance (Colo320R and HT-29R). All groups were cultivated in specific media, for the chemoresistant cells the culture media being supplemented with L-OHP 0.87 μg/ml at every second passage.

### Morphology analysis

The morphological analysis of the cells was performed with a light microscope (Carl Zeiss MicroImaging GmbH, Gottingen, Germany), with digital photographic capability. The microscopic images of cells were compared with respect to morphological characteristics (shape, polarity, intercellular distances and presence of pseudopodia).

### Evaluation of L-OHP cytotoxicity

The MTS/PMS (Promega Corporation, Madison, WI, USA) colorimetric cell proliferation assay was used for the Colo320 and Colo320R suspension-type and MTT test (Sigma-Aldrich, St. Louis, MO, USA) for HT-29 and HT-29R adherent cells, in order to simplify the solubilization procedures for the suspension-type cell lines [65]. Briefly, cells were seeded in triplicate in 96-well flat-bottom plates, at a cell population density of 15x10^3^ and 2x10^4^ for MTS/PMS and MTT assays, respectively. After 24 h, variable concentrations of L-OHP (0.001-300 μg/ml) were added and the cells were incubated for additional 24 h. Absorbances were recorded with an ELISA plate reader, at 490 nm wavelength (Tecan Sunrise, Grödig/Salzburg, Austria). The half maximal inhibitory concentration (IC_50_) values were calculated as the concentrations corresponding to a 50% reduction of the cellular growth.

### Evaluation of cross-links formation

Platinum-DNA cross-links induced by L-OHP on the selected cell lines were determined indirectly with CA. Single strand-breaks were induced in the L-OHP-treated DNA via ionizing radiation as secondary genotoxic agent. We evidenced the L-OHP-DNA cross-links by quantifying the reduction in the single-strand breaks provoked by the ionizing radiations subsequent to L-OHP treatment. Briefly, the selected cell groups were seeded in triplicate in 24-well plates at a cell population density of 4x10^5^. After 24 h incubation, cells were either irradiated with doses of 2 Gy or 4 Gy gamma radiations using a Co^60^ source (Theratron 1000, Theratronics, Inc., Ottawa, Ontario, Canada), or exposed first to L-OHP (50 or 100 μg/ml) for 2 h and afterward gamma-irradiated. Dose rate of the applied radiation source was 1, 98 Gy/min. The cells were transported from the site of the irradiation to the CA laboratory on ice, in order to avoid DNA repair process. CA was performed under alkaline conditions according to Tice’s protocol [66]. The images of the cell’s nuclei were evaluated for DNA migration (the level of strand breaks) using a fluorescence microscope (Nikon Eclipse E600, Tokyo, Japan), equipped with 510–560 nm excitation and 590 nm barrier filters. 200 cells from each group were scored and assigned to different lesion classes using the Collins’ classification method [67]. For each group a lesion score (LS) was calculated, based on the number of the cells assigned to each comet classes. LS was expressed as arbitrary units (AU) and the detailed description of the calculation of LS was made elsewhere [11].

### Microarray expression profiling

The gene expression profiling of the parental (Colo320 and HT-29) and L-OHP-resistant (Colo320R and HT-29R) cell lines was assessed with 4x44k Whole Human Genome Oligo Microarray G4112F slides (Agilent Technologies, Santa Clara, CA, USA) using two-color design. Three biological replicates of each cell lines, both parental and resistant, were tested in order to identify genes implicated in the L-OHP-resistance acquiring process. Total RNA was isolated using TriReagent (Sigma-Aldrich, St. Louis, MO, USA) and purified with RNeasy Mini Kit (Qiagen, Hilden, Germany). The quality of RNA was assessed with Bioanalyzer 2100 (Agilent Technologies, Santa Clara, CA, USA). All samples with an RNA integrity number (RIN) greater than 9 and an rRNA 28S/18S ratio higher than 1.8 were considered suitable for further analysis. The microarray probes were synthesized from 200 ng of total RNA and labelled with fluorochromes Cy3 and Cy5 using Low Input Quick Amp Labeling Kit (Agilent Technologies, Santa Clara, CA, USA), according to the manufacturer’s instructions. Technical replicates of each sample were used for hybridisation control (dye-swap design), each sample being labelled with Cy3 and Cy5 in independent experiments. In each array, RNA extracted from L-OHP-resistant cells was compared with RNA extracted from parental cells. Subsequently, all slides were scanned with Agilent Technologies scanner G2505BUS45102867 and quantification of microarray images were done with Feature Extraction software v. 10.5.3 (Agilent Technologies, Santa Clara, CA, USA).

### Microarray analysis

Microarray data analysis was performed in R statistical programming language [68]. Background and foreground intensity ratios were computed taking log2 ratios of intensities for red (R) and green (G) fluorescence channels (M values). No background subtraction was applied due to the weak correlation between background and foreground intensity ratios (ρ < 0.08). Within-array normalization was carried out using Loess regression. Data were further subjected to between-array normalization by quantile method. Median M values (log2(R/G) values) for duplicate spots were computed and used in class comparison analysis to identify changes in gene expression profiles involved in L-OHP-resistance acquiring process of Colo320 and HT-29 cell lines.

### Quantitative real-time PCR (qRT-PCR)

The expression levels of the genes selected by microarray were re-evaluated by qRT-PCR using Light Cycler 480 (Roche Applied Science, Penzberg, Germany) with primers (1 μM) and Universal Probe Library (UPL) probes (0.2 μM). *In silico* design of UPL probes and primers were obtained from Roche Applied Science Software as follow: PTPRO: F-ctatggagacatcactgtggaga, R-tcctgcatctcgtcagca (UPL#6); KRT18: F-tgatgacaccaatatcacacga, R-ctgggcttgtaggcctttta (UPL#63); NDRG1: F-gggtgcagaagggactagg, R-tgctcctggacatcaaactct (UPL#22); ID1:F-gctgctctacgacatgaacg, R-ctcaccttgcggttctgg (UPL#22); WIF1: F-ccagggagacctctgttcaa, R-ttgggttcatggcaggtt (UPL#76); AVEN F-ggtggtccaagaggaagaagt, R-gaaatcatgctgtccaacca (UPL#22); TGFB1 F-gcagcacgtggagctgta, R-cagccggttgctgaggta (UPL#72); MDK: F-ctcttagcggatgcagcac, R- ccgcccttcttcaccttatc (UPL#63); CYR61: F-aagaaacccggatttgtgag, R-gctgcatttcttgcccttt (UPL#66); 18 s rRNA: F-gcaattattccccatgaacg, R-gggacttaatcaacgcacgc (UPL#48). Each reaction was performed in 5 μl of 1:10 (v/v) dilution of the first cDNA strand synthesized with First Strand cDNA Synthesis Kit (Roche Applied Science, Germany) from 1 μg of the total RNA. The cDNA was then amplified with the Light Cycler Taqman Master Kit (Roche Applied Science, Germany) in a final volume of 20 μl. Thermal cycle conditions included 10 minutes at 95°C for enzyme activation followed by 40 cycles of 15 seconds at 95°C, 20 seconds at 55°C and 1 second at 72°C for the amplification step and 30 seconds at 40°C for the cooling step. The fluorescent signals of UPL probes were used to calculate the cycle thresholds. The target genes were normalized to 18S rRNA housekeeping gene and quantified using the comparative threshold cycle (2-ddct) method described by Livak and Schmittgen [69].

### Statistical analysis

Statistical processing of the cytotoxicity, CA and qRT-PCR results were done using GraphPad Prism software program, version 5.0 (San Diego, CA, USA). Statistical comparison between groups were made by one-way Anova and Bonferroni posttest and by unpaired two-tailed *t* test for qRT-PCR data (p < 0.05). For the microarray experiment, the correlation between background and foreground intensity ratios (M values) was assessed in R using Spearman’s rank correlation test. Differentially expressed (DE) genes between resistant and parental cell lines were selected with Limma package/R by fitting a linear model to the expression data for each gene and using empirical Bayes methods to moderate the standard errors across genes [70]. A gene was considered differentially expressed if M value was lower than -0.58 or greater than 0.58 (at least 1.5 -fold down- or up-regulation in resistant versus parental cells) and p value adjusted for multiple testing < 0.05 (Benjamini and Hochberg method). Pearson correlation between microarray and qRT-PCR results were performed in GraphPad Prism software program, version 5.0 (San Diego, CA, USA).

### Functional analysis

Functional profiling was performed using Ingenuity Pathway Analysis (IPA) software (Ingenuity Systems, Redwood City, California) [64]. Accession numbers of DE genes associated with M values were uploaded into the software. Using information stored in the Ingenuity Knowledge Base (IKB), genes were mapped to genetic networks, molecular functions and canonical pathways. The significance of the association between the genes and the molecular functions and the canonical pathways was determined by Fischer’s exact test (p < 0.05). IPA Upstream Regulator Analysis was used to identify key molecules (upstream regulators) which can affect the expression of their target genes and can regulate each other. To predict the activation state of the upstream regulators (“activated” or “inhibited”), a z-score was computed for each of them. The terms “activated” or “inhibited” does not necessary mean that the regulator is literally activated respectively inhibited. An “activated” upstream regulator indicates a molecule expected to be more active in the resistant cell lines than in the parental ones. A p-value less than 0.01 and a z-score greater than 2 (prediction state: “activated”) or smaller than -2 (prediction state: “inhibited”) were considered significant.

## Competing interests

The authors declare that they have no competing interests.

## Author’s contributions

PV: conceived, designed and coordinated the study; induced chemoresistance in the tested cell lines; performed the morphology analysis; EFF: performed comet assay; MPS: read and interpreted the comet assay results; drafted the manuscript; IB: participated in the design of the study and performed statistical analysis for the cellular studies; CT: performed cytotoxicity assays; prepared and treated the cells for further studies; BV: irradiated the cells with gamma radiations. LB: performed statistical and bioinformatic analysis of microarray data; IB-N: carried out the RT-PCR study; OB: carried out the microarray study; participated in the study’s design and drafted the manuscript. All authors read and approved the final manuscript.
